# WPI Hydrogels with a Prolonged Drug-Release Profile for Antimicrobial Therapy

**DOI:** 10.3390/pharmaceutics14061199

**Published:** 2022-06-04

**Authors:** Valentina O. Plastun, Ekaterina S. Prikhozhdenko, Olga I. Gusliakova, Svetlana V. Raikova, Timothy E. L. Douglas, Olga A. Sindeeva, Oksana A. Mayorova

**Affiliations:** 1 Science Medical Center, Saratov State University, 83 Astrakhanskaya Str., 410012 Saratov, Russia; prikhozhdenkoes@sgu.ru (E.S.P.); olga.gusliakova17@gmail.com (O.I.G.); 2 Department of Microbiology, Virology and Immunology, Saratov State Medical University Named after V. I. Razumovsky, 112 Bolshaya Kazachia St., 410012 Saratov, Russia; matiz853@yandex.ru; 3 Saratov Hygiene Medical Research Center of the FBSI “FSC Medical and Preventive Health Risk Management Technologies”, 1A Zarechnaya Str., 410022 Saratov, Russia; 4 Engineering Department, Lancaster University, Gillow Av., Lancaster LA1 4YW, UK; t.douglas@lancaster.ac.uk; 5 Materials Science Institute (MSI), Lancaster University, Gillow Av., Lancaster LA1 4YW, UK; 6 Center for Neurobiology and Brain Restoration, Skolkovo Institute of Science and Technology, 3 Nobel Str., 143005 Moscow, Russia; o.sindeeva@skoltech.ru; 7 Department of Botany, Chemistry and Ecology, Saratov State Vavilov Agrarian University, 1 Theater Square, 410012 Saratov, Russia

**Keywords:** antimicrobial activity, hydrogel, drug release, cefazolin, whey protein isolate

## Abstract

Infectious sequelae caused by surgery are a significant problem in modern medicine due to their reduction of therapeutic effectiveness and the patients’ quality of life.Recently, new methods of local antimicrobial prophylaxis of postoperative sequelae have been actively developed. They allow high local concentrations of drugs to be achieved, increasing the antibiotic therapy’s effectiveness while reducing its side effects. We have developed and characterized antimicrobial hydrogels based on an inexpensive and biocompatible natural substance from the dairy industry—whey protein isolate—as matrices for drug delivery. The release of cefazolin from the pores of hydrogel structures directly depends on the amount of the loaded drug and occurs in a prolonged manner for three days. Simultaneously with the antibiotic release, hydrogel swelling and partial degradation occurs. The WPI hydrogels absorb solvent, doubling in size in three days and retaining cefazolin throughout the duration of the experiment. The antimicrobial activity of cefazolin-loaded WPI hydrogels against *Staphylococcus aureus* growth is prolonged in comparison to that of the free cefazolin. The overall cytotoxic effect of cefazolin-containing WPI hydrogels is lower than that of free antibiotics. Thus, our work shows that antimicrobial WPI hydrogels are suitable candidates for local antibiotic therapy of infectious surgical sequelae.

## 1. Introduction

Reconstructive and restorative surgery is commonly accompanied by a bacterial infection, which is a serious complication and can lead to poor treatment outcomes. Severe traumatic edema, circulatory disorders, accumulation of blood and fluid, and damaged dead tissues cause the development of various forms of wound infection [1]. In most cases, infectious contaminations are accompanied by repeated hospitalization and may require additional surgical intervention. This leads to both recovery time and positive results requiring increased time after the surgery [2,3,4,5]. The classic treatment for infectious complications after surgical procedures is systemic antibiotic therapy. Since normal doses of antibiotics in their systemic use may be insufficient for local bactericidal action in the area of infection, it is necessary to increase the administered doses. Prolonged systemic use of antibiotics can lead to the development of unwanted side reactions, increasing the number of toxic effects [6,7,8]. The combination of these factors leads to the ineffectiveness of antibiotic therapy. Despite the improvement of treatment methods and surgical techniques and the emergence of new highly active antibiotics, the recurrence rate remains high. The use of various systems to deliver antibiotics to the site of infection has become a common method of local suppression that continues to improve [9,10,11,12]. Over the years, several options for silver-based [13] and iodine-based [14] antibacterial coatings have been developed to reduce the risk of postoperative infections after implantation. However, such technologies are currently not yet available on the market and/or have limitations in use, such as high drug toxicity [15,16].

Another promising material for local infection suppression is hydrogel. At present, the use of hydrogels for biological applications is being carefully studied due to their structural similarity to natural extracellular matrices [17]. Biocompatibility and non-toxicity are the main characteristics of hydrogels for their use in regenerative medicine [18]. A hydrogel coating based on hyaluronic acid and poly-D,L-lactide has recently been developed [DAC^®^ (Defensive Antibacterial Coating), Novagenit Srl, Mezzolombardo, Italy]. This hydrogel is expected to be useful as an antibacterial coating for implantable devices that provides a protective barrier against bacterial adhesion [19,20,21,22]. Clinical trials have demonstrated its ability to reduce postoperative infectious sequelae associated with implants [23,24]. Currently, the data on its safety and efficacy in use in patients is low. The main disadvantage of the DAC^®^ gel is the limited loading capacity of the hydrogel, as well as the lack of in vitro studies of the antimicrobial activity of the gel against infection. In addition, the high cost of using a single 10 mL gel syringe remains a critical factor limiting its usage in medical practice.

The whey protein isolate (WPI) used in this work is a by-product of the processing of whey used in cheese preparation [17,25]. In this regard, WPI is a cheap and available product that is used as an emulsifier and as gelling, foaming, and water-binding agents in the food, pharmaceutical, and cosmetic industries [17,25,26,27,28,29]. It is known that the composition of WPI can vary depending on the method of isolating the WPI [30,31]. The HPLC analysis of the WPI composition, carried out in works [32,33], showed that the protein content in lyophilized WPI powder is about 96% (70% of β-lactoglobulin, 26% of α-lactalbumin); bovine serum albumin is a minor component of WPI and is about 4%. Gelation occurs due to the denaturation of the native protein β-lactoglobulin after an increase in temperature [34,35]. The main advantage of protein-based delivery systems is the non-toxicity and biocompatibility of the initial components. It is known that whey protein reduces the pro-inflammatory risks and increases the level of anti-inflammatory cytokines, thereby promoting the healing of skin wounds [36]. The ability of WPI hydrogels to withstand autoclave sterilization and to provide the controlled release and high bioavailability of hydrophobic drugs are primary benefits of WPI cross-linked hydrogels over other materials for local suppression and prevention of postoperative infections [29,37,38]. Several important characteristics of hydrogels as potential scaffolds in regenerative surgery are their strength and thermal and mechanical properties. Previously, it was shown that the destruction of WPI-based hydrogels occurs at temperatures above 250 °C, which leads to the chemical decomposition of the WPI molecules. Heating to temperatures below 250 °C results in the loss of water adsorbed in hydrogels without the WPI bond opening [39,40,41]. The mechanical properties of WPI hydrogels depend on the material porosity; smaller pore sizes and a decrease in the frequency of their distribution lead to an increase in the Young modulus of elasticity and, as a result, an improvement in the physical characteristics of the hydrogels [42]. At the same time, the addition of components capable of forming additional cross-links to the hydrogels leads to an increase in their physical strength [42,43].

We have proposed a biocompatible long-acting hydrogel for the prevention of bacterial infections based on a natural protein preparation, whey protein isolate (WPI), with the addition of the antibacterial drug cefazolin. WPI hydrogels are able to fill wound spaces and provide higher concentrations of antibacterial drugs in comparison to systemically administered doses after surgery [20,44,45,46]. Histological analysis of mouse muscle tissues ex vivo showed that the subcutaneous implantation of biofilms based on whey protein in vivo does not cause necrosis or degradation of the surrounding tissues or the release of exudate in the area of interest, which confirms the non-toxicity and immunogenicity of WPI hydrogels [47].

In the present research, we studied the kinetics of the release of an antibacterial drug and the effect of a solvent on the swelling capacity and degradation of hydrogels with different antibiotic concentrations in vitro. A quantitative evaluation of the antibacterial effect of free cefazolin and hydrogels with different antibiotic concentrations, contained in the pores of WPI, was carried out following two different methods against *Staphylococcus aureus* using flow cytometry and fluorescence spectroscopy. The effect of antibacterial WPI hydrogel disks on the overall survival and proliferation of L929 fibroblast cells was studied in comparison with a solution of this antibiotic.

## 2. Materials and Methods

### 2.1. Materials

Eagle’s Minimum Essential Medium (1X) and Fetal Bovine Serum were obtained from Gibco (Paisley, UK). AlamarBlue (Cell Viability Reagent) was obtained from Invitro-gen (Waltham, MS, USA). Sodium chloride (Ph. Eur., pure, pharma grade) was obtained from PanReac AppliChem (Darmstadt, Germany). Propidium iodide (PI) and Syto 9 were purchased from Thermo Fisher Scientific (Eugene, OR, USA). DAPI and Calcein AM was purchased from Sigma Aldrich (Steinheim, Germany). WPI (BiPRO, Davisco Foods Int., Inc., Eden Prairie, MN, USA) with 97.7% protein and 75% β-lactoglobulin in dry matter (according to the specification) was used without further purification as described previously [29,30,33]. Cefazolin (powder for the preparation of a solution for intravenous and intramuscular administration) was purchased from PJSC Krasnfarma (Krasnoyarsk, Russia) and used without prior purification. Mueller-Hinton Agar No. 2 was obtained from HiMedia Laboratories (Mumbai, India). Other reagents used in the investigation were purchased from Sigma-Aldrich (Steinheim, Germany). Millipore Milli Q water (18.2 M·cm${}^{-1}$) was used as an aqueous medium during all sets of experiments.

### 2.2. Fabrication of WPI-Based Hydrogels

The WPI-based hydrogels were prepared by thermo-induced curing. At the first stage, a stock solution containing 40% WPI was prepared. To complete this, dry WPI powder was dissolved in deionized water, thoroughly mixed, and left for 8–12 h for the foam to settle. Then, the solution was poured into four 2 mL Safe-Lock Tubes, sodium cefazolinate powder was added to three samples at the rate of 0 mg (control), 0.5 mg, 5 mg, and 10 mg per 1 mL of WPI solutions, respectively. Next, all solutions in the tubes were heated for 15 min in an oven at 90 °C to solidify the samples. The hardened and cooled samples were removed from the tubes, cut into disks 1 mm thick, 9 mm in diameter, weighed, and autoclaved.

### 2.3. Water Swelling and Cefazolin Release of WPI-Based Hydrogel Samples

The swelling character of the hydrogel samples was studied in saline (0.9% NaCl). To measure the swelling, after autoclaving, hydrogel samples with known weight were placed in 2 mL Eppendorf tubes containing 1 mL of saline and incubated at 37 °C under constant stirring (300 rpm) from 15 min to 144 h (6 days). The swollen gels were periodically (0.5 h, 1 h, 3 h, 6 h, and up to 144 h) removed, blotted on dry filter paper to remove excess water, and immediately weighed. Then, the mass increase (*MI*) was calculated as:

$$MI\left(\%\right)=(\left(M_{t}-M_{0}\right)/M_{0})\times 100\%,$$

where $M_{t}$ is the weight of the hydrogel at a certain time, and $M_{0}$ is the initial hydrogel weight.

For the cefazolin release, an aliquot of each incubated WPI hydrogel sample was selected, and the optical densities of the solutions were measured using a CLARIOstar Plus microplate reader (BMG Labtech, Ortenberg, Germany) and a Greiner UV-STAR^®^ MICROPLATE (Greiner Bio-One, Kremsmünster, Austria) in the 250–650 nm range (1 nm step). The optical density of the 281 nm line (absorbance maximum of cefazolin) was used in the calibration and release amount estimation. The calibration trend line was calculated using the linear fit of 281 nm line mean values at different concentrations. All experiments were carried out with *n* = 5.

### 2.4. Scanning Electron Microscopy

For Scanning Electron Microscopy (SEM) imaging, the hydrogel samples were preliminarily freeze-dried 24 h after the cooling period. Freeze-dried disks of the WPI hydrogel were fixed on a Si substrate using double-sided electrically conductive carbon tape, and then the samples were sputtered with gold in a vacuum. Then, SEM measurements were performed with a MIRA II LMU (TESCAN) microscope at an operating voltage of 30 kV. All measurements were performed at the Educational and Scientific Institute of Nanostructures and Biosystems of the Saratov State University.

### 2.5. In Vitro Study

#### 2.5.1. Bacterial Tests

For this study, *Staphylococcus aureus* ATCC 29213, received from Saratov State Medical University Microbiology, Virology, and Immunology Department collection, was taken as a test culture. The antimicrobial activity study was carried out using standard methods [48]. To assess the prolonged inhibition of bacterial growth on the surface of a solid medium, the agar diffusion method was used. The 90 mm Mueller-Hinton agar plates were inoculated with *S. aureus* suspension (1.5 × 10${}^{8}$ CFU/mL) and left to absorb for 15 min. WPI-based hydrogel samples with cefazolin, control WPI samples, and control paper disks with cefazolin were placed on the agar surface and incubated at 37 °C. Microbial growth inhibition zones were recorded every 24 h, and all samples were transferred to fresh agar plates.

In parallel, the antibacterial effect of cefazolin-containing hydrogels in a liquid medium was studied. Each hydrogel sample was placed in a 16 mm glass tube with Mueller–Hinton nutrient broth (1 sample per 1 mL) and inoculated with *S. aureus* suspension (resulting amount of bacteria 5 ×10${\;}^{5}$ CFU/mL). All tubes were incubated at 37 °C, each of the 24 optical densities of the broths was measured using DEN-1B densitometer (Biosan, Latvia). Then, the samples were moved into fresh tubes with inoculated broth, and all procedures were repeated for up to 72 h. All experiments were carried out with *n* = 5.

The identification of live and dead bacteria was performed using the standard protocol [49]. After incubation with the hydrogels, *S. aureus* suspensions were carefully prepared by washing–centrifugation in saline three times. After the final washing stage, the cell pellets were resuspended in 1 mL of saline and incubated with 1 μL Syto 9 (5 mM/mL) and 20 μL of propidium iodide (1 mg/mL) at room temperature in the dark for 15 min. Syto 9 and PI were used to visualize the live and dead bacteria, respectively.

#### 2.5.2. Cell Culturing

Mouse fibroblasts (L929 cell line) were used for toxicity tests. L929 were cultured in Eagle’s Minimum Essential Medium supplemented with a 10% fetal bovine serum without any antibiotics (complete growth media) in a humidified incubator containing 5% CO_2_ at 37 °C. The subculturing procedure was carried out according to ATCC protocol.

Cells were seeded in 48-well plates at a density of 25,000 cells per well. The following day, the excised hydrogel disks were added to wells after renewing the media. Subsequently, the cells were incubated (Innova CO-170, New Brunswick Scientific, Enfield, CT, USA) at 37 °C for 48 h, together with the added materials. In the last step, the hydrogel disks and media were removed from the wells and discarded, 300 μL of the fresh media was added to each well with following adding of 30 μL of AlamarBlue. The cells with the hydrogels and their intensities were measured using an ultraviolet-visible spectrometer Synergy H1 Multi-Mode Reader (BioTek Instruments, Inc., Winooski, VT, USA). The experiment showed the capability of metabolically active cells to convert the AlamarBlue reagent into a fluorescent and colorimetric indicator [50]. To establish the irreversibility of the effect of hydrogels and cefazolin on L929, all disks were discarded, and the culture medium was replaced with a new one without antibiotics after 48 h of incubation. All experiments were carried out with *n* = 4.

### 2.6. Confocal Laser Scanning Microscopy (CLSM)

To perform the live/dead analysis of *S. aureus*, a suspension of 600 μL was added to μ-Slide 4 Well Glass Bottom (Ibidi, Gräfelfing, Germany) after staining and was studied with a Leica TCS SP8 X inverted confocal microscope (Leica Microsystems, Wetzlar, Germany) equipped with a diode (405 nm) and argon-pulsed laser sources. *S. aureus* samples were visualized with a 488 nm laser line of an argon laser source focused through a 20×/0.70 N.A. objective. Emission detection ranges were 500–520 nm (green, Syto 9, live bacteria) and 650–725 nm (red, PI, dead bacteria).

L929 cells were seeded to Nunc™ Lab-Tek™ II Chambered Coverglass (4 wells) at the density of 100,000 cells per well. The following day, the excised hydrogel disks were added to the wells after renewing the media. Subsequently, the cells were incubated at 37 °C for 48 h together with the added materials.

For the morphological analysis of the cells after incubation, a stock solution based on the complete growth media containing DAPI (10 μg/mL) and Nile Red (5 μg/mL) was prepared. Preliminary, the Nile Red solution in DMSO was prepared at a concentration of 1 mg/mL.

Before staining, the hydrogel disks were removed from the wells, and the media from each well was aspirated. The layer of cells was carefully washed twice with DPBS. For staining, 500 μL of stock solution was added to each well, and the cells were incubated for 20 min in a humidified incubator containing 5% CO_2_ at 37 °C. Subsequently, the staining solution was aspirated off and each well was washed twice with DPBS. Then, a new complete media was added to the wells.

CLSM images of the stained L929 cells were obtained with Leica TCS SP8 X. DAPI was visualized with 405 nm excitation, 420–495 nm emission detection range (blue channel), and Nile Red was visualized with excitation using 514 nm laser line of argon source, 540–620 nm emission detection range (red channel). Laser sources were focused through 20×/0.70 N.A.

### 2.7. Flow Cytometry

Live and dead bacteria were counted and evaluated using the imaging flow cytometer Amnis ImageStream X Mk II (Luminex, Austin, TX, USA). Fluorescent dyes were excited by a 488 nm and 561 nm laser at 100 mW power. Flow cytometry data were processed using IDEAS software (Luminex, Austin, TX, USA).

### 2.8. Statistical Analysis

The statistical data on the WPI hydrogels’ swelling, both with and without cefazolin, the cefazolin release, average growth inhibition zone sizes of *S. aureus*, and the cytotoxic activity of the hydrogels were calculated using Microsoft Excel. Means and standard deviations were obtained from five independent experiments in each case.

## 3. Results and Discussion

### 3.1. Preparation and Characterization of Antibacterial WPI Hydrogels

**Figure 1:**
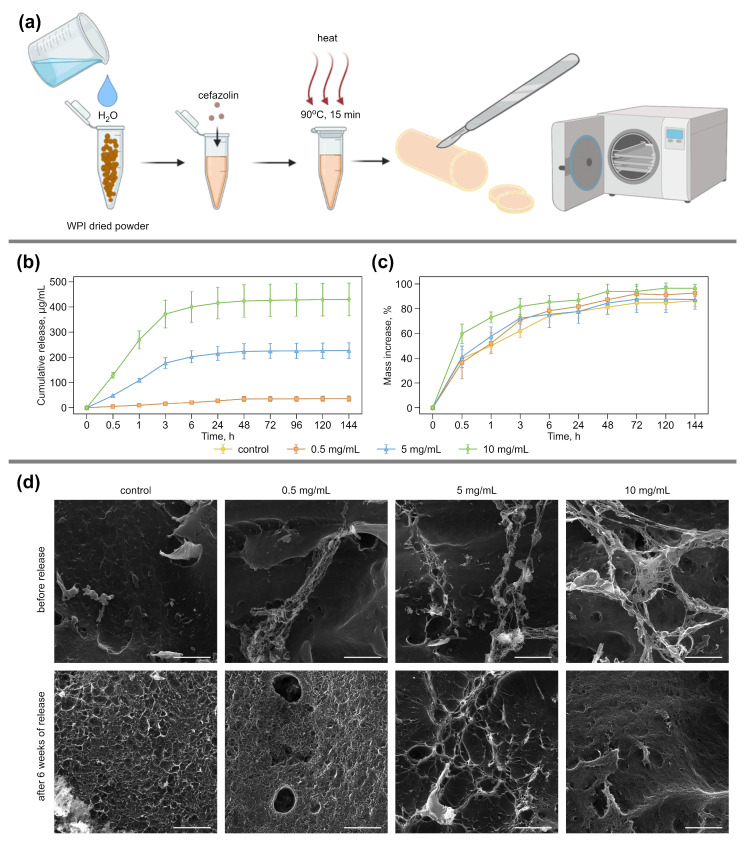
(**a**) The principal scheme for the formation of WPI-based hydrogels containing cefazolin. Control WPI hydrogels were prepared similarly without the addition of antibiotics. (**b**) The cefazolin cumulative release profile from WPI hydrogel disks incubated in saline at 37 °C for 144 h. Error bars show cumulative standard deviations calculated from five measurements for a sample in each treatment stage. (**c**) Mass increase (swelling profile) of WPI hydrogels containing cefazolin incubated in saline at 37 °C. Error bars show standard deviation, calculated from five measurements for a sample in each treatment stage. (**d**) Morphological analysis of WPI hydrogels with cefazolin. The scale bar for SEM images of transverse-sections is 5 μm.

WPI hydrogels containing the antibacterial drug cefazolin at various concentrations (0; 0.5; 5; 10 mg/mL) were prepared via the heat treatment of 2 mL of an aqueous solution of WPI (40 mg/mL) with the addition of the required amount of antibiotics [51]. In the present study, WPI with a known composition was used. According to the manufacturer’s specification, the WPI contained β-lactoglobulin (75.7 ± 1.4%), α-lactalbumin (14.7 ± 0.1%), and <4% BSA (BiPRO, Davisco Foods Int., Inc., Eden Prairie, MN, USA). It is known that the heat treatment of a WPI solution above 60 °C promotes the gelation process due to protein denaturation and the formation of new disulfide bonds with the formation of a three-dimensional network [52,53,54,55]. To remove unwanted biofilms from the surface of the formed hydrogels, which can provoke additional bacterial contamination, all samples were additionally sterilized by autoclaving at 121 °C for 15 min (Figure 1a). In medical practice, cefazolin is most often used as an antibiotic against a wide range of bacteria [56,57,58]. Hence, this antibiotic was used for our research.

The main advantage of hydrogels prepared by solution heat treatment is their loading capacity. The addition of cefazolin into the initial solution before polymerization makes it possible to obtain hydrogels containing a known amount of the drug without loss. The incorporation of cefazolin molecules into the pores of the hydrogel occurs during the formation of disulfide bonds and the formation of a three-dimensional hydrogel structure [59,60]. It was previously shown that the addition of small-molecule drugs to the WPI solution does not affect the nature of the protein binding during the formation of hydrogel porous structure [51].

Our study was aimed at studying the release rate of cefazolin from hydrogels. The release of cefazolin from WPI hydrogels (Figure 1b) was carried out in saline at 37 °C for 144 h (6 d). As can be seen from the graph, the diffusion of low molecular weight cefazolin from the pores of the hydrogel begins from the first minutes after the disk is immersed in the solution and occurs within 72 h. The maximum amount of cefazolin released from the hydrogels is observed 6 h after immersion in an aqueous solution. After 96 h, antibiotic release has stopped for all samples of WPI hydrogels with different levels of cefazolin.

The cefazolin release from the WPI hydrogels occurs under the action of solvent diffusion and directly depends on their swelling degree [61]. The swelling capacity of hydrogels depends on its elasticity and the ability to stretch polymer chains and, thus, increase the hydrogel mass by moisture absorption [35]. A swelling test was performed onWPI hydrogels with various cefazolin contents (0; 0.5; 5; 10 mg/mL) in saline at 37 °C for 144 h (6 d) simultaneously with the cefazolin-release test (Figure 1c). All WPI hydrogels showed a 50% mass increase (MI, %) after 30 min of incubation. After 48 h, the increase in the mass was about two times; the absorbed solvent amount in the gel structure remained unchanged until the end of the experiment. The MI values for all samples were within the statistical error, indicating that there is no covalent bond between the antibiotic and the protein, which reduces the degree of swelling [62,63].

In parallel with the processes of swelling and cefazolin release from the hydrogel pores, a partial degradation of the hydrogels occurs. This was shown on the SEM images 6 weeks after the incubation of the WPI hydrogel disks with cefazolin in saline at room temperature (Figure 1d). The long-term storage of hydrogels in physiological saline at room temperature leads to their slight degradation. For samples with the lowest content of the antibiotic (0 mg/mL and 0.5 mg/mL), the formation of a porous structure of the hydrogel surface is observed compared to the surface of the same native hydrogels prior to saline incubation. This indicates a partial degradation of the upper layer of the hydrogel disks. For hydrogels with high concentrations of cefazolin (10 mg/mL), surface damage is insignificant. It is known that WPI is a natural component with biocompatibility and biodegradation properties [64,65], which is confirmed by SEM imaging data. Sustained release over several days, moisture retention, and biodegradability are the necessary qualities of hydrogels for use in the prevention and treatment of postoperative infection as a drug delivery system.

### 3.2. Antibacterial Effect

**Figure 2:**
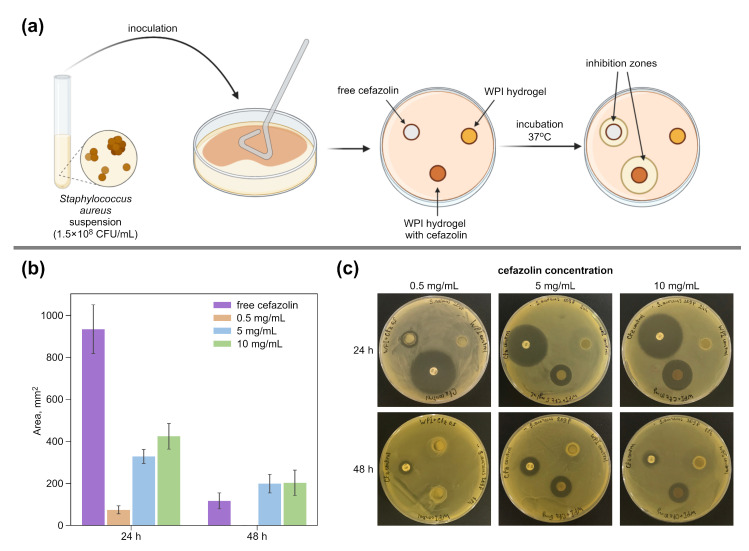
(**a**) The principal scheme of the experiment to study the antibacterial activity of WPI-based hydrogels with different cefazolin concentrations on dense nutrient media against *S. aureus*. (**b**) Average growth inhibition zone sizes and (**c**) visualization of *S. aureus* bacterial strains during study of an antimicrobial action of WPI-based hydrogels containing cefazolin, cefazolin standard disk (control), WPI-hydrogel (0 mg/mL of cefazolin) by agar diffusion method. Values are presented as means ± standard deviations (*n* = 5).

The antibacterial activity of WPI-based hydrogels against the opportunistic bacteria *S. aureus* was investigated by the disk diffusion method on a solid-nutrient medium (Figure 2a). Each of the hydrogel samples with cefazolin (0.5, 5 or 10 mg/mL), the control hydrogel sample without antibiotic, and the control paper disk with free cefazolin were placed on an experimental agar plate inoculated with *S. aureus* suspension (0.5 McFarland). Then, the experimental plates were incubated at 37 °C for 24 h. After that, the bacterial growth inhibition zones were measured on each plate, and the samples were transferred to fresh agar, inoculated with *S. aureus*. The experiment was repeated for up to 72 h. As can be seen in Figure 2b,c, all cefazolin-containing hydrogels retain their antimicrobial activity after the preparation procedure. After 48 h of the experiment, the samples containing 5 mg/mL and 10 mg/mL of cefazolin still retained the ability to inhibit bacterial growth. The difference between the growth inhibition areas of these samples is insignificant, which corresponds with their release profiles (Figure 1b). WPI gel samples with cefazolin do not show such a sharp decrease in activity as the free antibiotic control samples, which confirms their ability to have a prolonged antimicrobial effect.

**Figure 3:**
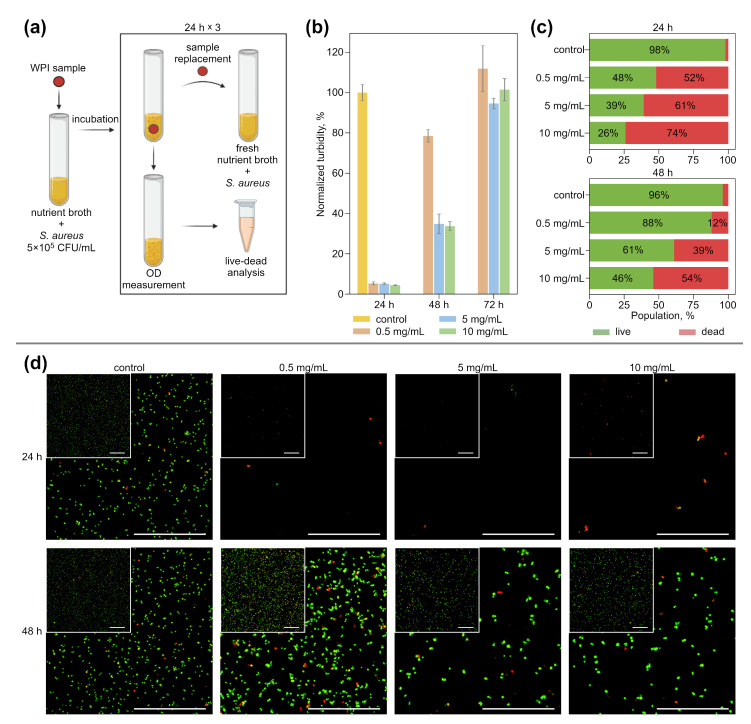
(**a**) The principal experiment scheme to study the antibacterial activity of WPI-based hydrogels with cefazolin in liquid media. (**b**) Total number of bacteria in nutrient broth compared with control (**c**) live–dead analysis of experimental bacterial suspension by flow cytometry. (**d**) CLSM images of bacterial suspensions after incubation with cefazolin-containing hydrogel samples. Green color indicates live bacteria (Syto 9), red color indicates dead ones (PI). The scale bar in all images is 50 μm.

In the next series of experiments, the ability of WPI-based hydrogels with cefazolin to inhibit the *S. aureus* growth in a liquid nutrient medium was studied (Figure 3). Hydrogel samples with an antibiotic in three studied concentrations and control hydrogel samples were placed in a nutrient broth, inoculated with *S. aureus*, and incubated at 37 °C (Figure 3a). Every 24 h, the turbidity of the nutrient broth was measured to determine the amount of CFU. Then, the aliquots of nutrient broth from these tubes were taken for visualization via live–dead analysis. Samples from the tubes where bacterial growth was not detected or was lower than the control were transferred to the tubes with fresh nutrient broth with *S. aureus* and incubated again. The experiment lasted 72 h. After the first 24 h, all hydrogel samples with cefazolin inhibited *S. aureus* growth (Figure 3b). Hydrogels containing 5 mg/mL and 10 mg/mL of cefazolin kept this effect up for up to 48 h of the experiment.

Live–dead analysis by flow cytometry showed (Figure 3c) that the use of hydrogels at any concentration provided more than 50% dead cells in the population in the first 24 h. Dead cells are understood as objects with a predominance of the PI signal over Syto 9. As noted in [66,67,68], the population of cells simultaneously positive for PI and Syto 9 (or any other fluorescent dye characterizing cell viability) has been considered as injured or sub-lethal. It means, effectively, that the membrane of bacterial cells of this population was already disturbed significantly at the time of the measurements. A cell population with a strong predominance of PI over Syto 9 demonstrates significant membrane damage, due to which a large amount of PI was able to penetrate into the bacterial cell.

Figure 3b shows the total number of bacteria present in the measured sample, while Figure 3c shows the percentage composition of live and dead cells in the populations. As can be seen in Figure 3b,c, hydrogels with 5 mg/mL and 10 mg/mL cefazolin not only provide a significant reduction in the number of bacteria in the sample, but also damage more than 60% of the bacterial cells in this reduced amount. Moreover, the use of a hydrogel with an antibiotic at a concentration of 10 mg/mL provides damage to more than 50% of the bacterial population even after 48 h of sample use. This indicates the high antimicrobial efficacy of the developed hydrogels.

CLSM images of bacterial suspensions incubated with test samples with cefazolin and control suspension are shown (Figure 3d) to visualize the antibacterial effect of hydrogels. The images confirm a substantial reduction in the number of bacteria in the suspension when incubated with hydrogels containing antibiotics (the volumes of the studied samples of bacterial suspensions were always chosen to be the same). The red staining of the bacteria is due to severe membrane damage and provides an idea of the ratio of bacterial sub-populations.

Thus, WPI hydrogels containing cefazolin in concentrations of 5 mg/mL and 10 mg/mL can inhibit the growth of *S. aureus* in liquid nutrient media over 48 h.

### 3.3. Impact of Hydrogels with Cefazolin on Eukaryotic Cells

**Figure 4:**
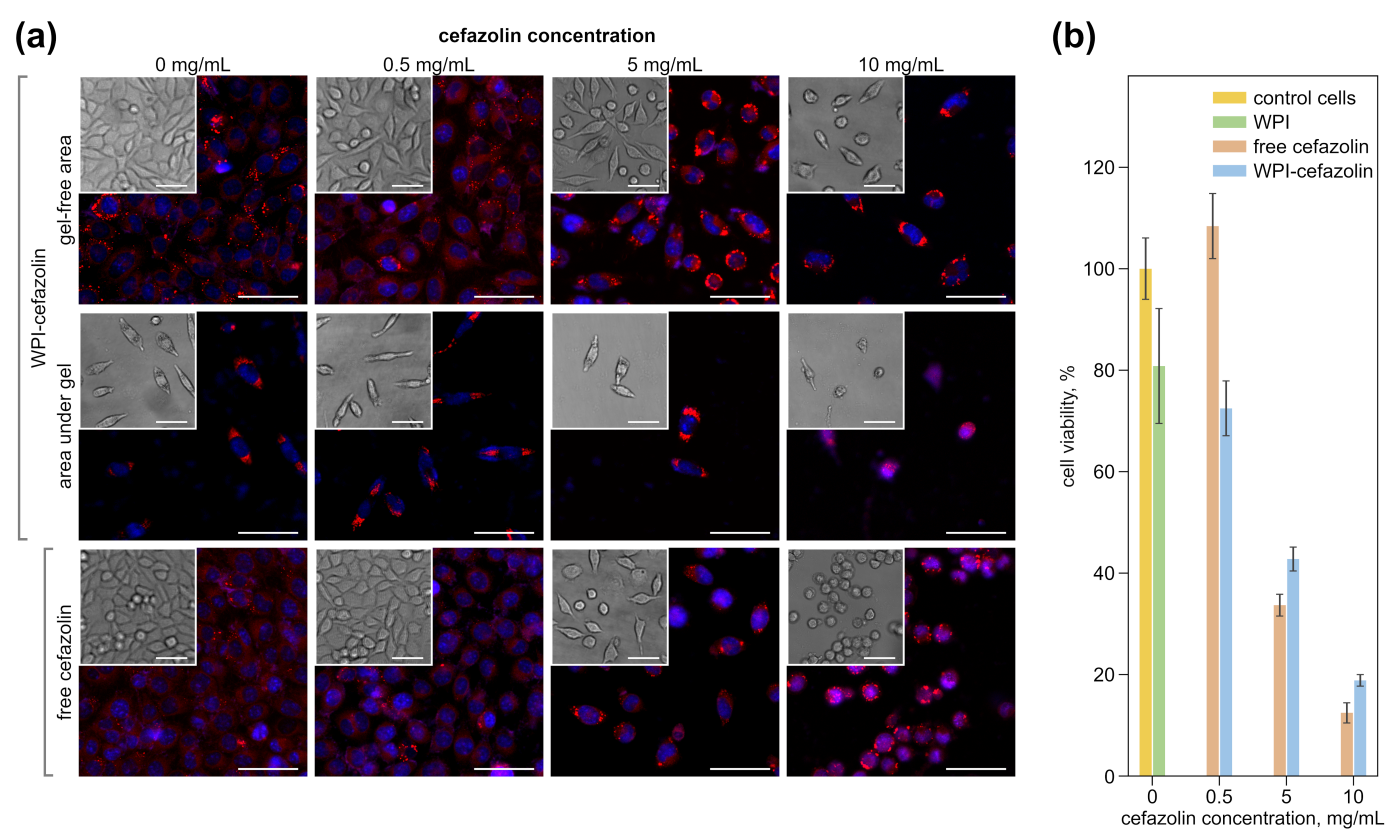
(**a**) CLSM images of L929 incubated with free and immobilized cefazolin (0, 0.5, 5, 10 mg/mL) for 48 h. For immobilized cefazolin, gel-free areas and areas under gel are specified. Blue color indicates nuclei (DAPI), and red indicates lipid components on membrane and inside the cell (Nile red). Images inserted to the left upper corner are brightfield images. Scale bars are 50 μm. (**b**) Viability of the L929 cell line incubated with free and immobilized in WPI disks cefazolin (0, 0.5, 5, 10 mg/mL) for 48 h.

The free antibiotic, as well as the antibiotic-containing, WPI hydrogels, have different effects on the morphology of a fibroblast cell line (L929) as was demonstrated with CLSM. Figure 4a shows both fluorescent and bright field images of cells after 48 h of incubation with free and immobilized-in-hydrogels cefazolin. Cell nuclei are blue (DAPI) and lipids located on the membrane and inside the cell are red (Nile red). Cells cultured without a free antibiotic and with an empty WPI disk are flattened and have a well-defined rounded nucleus, which corresponds to the normal cell state. With an increase in the concentration of cefazolin used, both in free form and encapsulated in hydrogels, the number of cells decreases in the view field compared to the control, since cefazolin inhibits the proliferation of epithelial-like cells [69]. At the minimum concentration of the antibiotic, the cells retain their elongated shape. There is a trend towards a decline in the intensity of Nile Red staining (a lipophilic dye) of cell membranes with the increasing concentration of antibiotics, both in free form and encapsulated in hydrogels. The staining of the intra-cellular lipid structures (lipid drops) remains intense. This effect may be associated with the influence of the cefazolin on the antioxidant system of a cells, which leads to an increase in reactive oxygen species inside the cells, which in turn causes lipid peroxidation in membranes [70]. However, when incubated with free cefazolin at a concentration of 10 mg/mL, all cells in the view field become rounded, detaching from the substrate, which indirectly indicates the negative effect of the antibiotic. In the case of using a hydrogel with cefazolin in a similar concentration, some of the cells remain spread and attached to the substrate. Thus, it can be concluded that the cytotoxicity of the antibiotic is reduced when it is encapsulated into a hydrogel. The microscopy of the cells incubated with the hydrogel disks revealed two regions differing in cell density (Figure 4a). In the area located under the disk, the cell density was significantly lower than in the area free from the hydrogel. This effect is most likely associated with the mechanical disruption of the cell layer due to the free floating of the disk in a liquid medium (see Section 2). We consider this result to be an example of the successful localization of the active substance action, which is necessary for the local delivery of antibiotics in the treatment of soft-tissue infection and osteomyelitis and the suppression of post-surgical infections [71].

To study the effect of the antibiotic-containing hydrogels on the viability of eukaryotic epithelial-like cells, a hydrogel and free cefazolin cytotoxicity assay was performed on L929 cells using Alamar Blue, which measures the metabolic activity of cells (Figure 4b). Cultivation of L929 cells for 48 h in the presence of WPI hydrogel disks without and with minimal antibiotic (WPI-hydrogel, 0.5 mg/mL WPI-Cefazolin) showed that the samples had little cytotoxic effect on the cell line. Cytotoxicity of not more than 20% was previously noted for both WPI hydrogels [51] and WPI micro- and nanoemulsions [72]. The low cytotoxicity may be associated with a decrease in the pH of the medium during the degradation of the hydrogel disks in the culture medium. It is known that whey proteins have an acidic pH [73,74]. Thus, WPI molecules can be released into a culture medium during prolonged incubation and lead to its acidification. Carla R. Kruse et al. showed that a slightly acidic environment (pH 6.5) leads to a minor decrease in fibroblast viability (down to about 80%) [75]. The percentage of cell viability decreased with a rise in the amount of antibiotic in the hydrogel disk. So, for hydrogels with 5 mg/mL cefazolin, cell survival was 43%. For hydrogels with 10 mg/mL cefazolin, the cytotoxic effect was more pronounced, the survival was less than 20%. The use of free cefazolin at high concentrations (5 mg/mL and 10 mg/mL) demonstrates a greater cytotoxic effect compared to the hydrogel-encapsulated form.

An experiment was also carried out to determine whether the effects of hydrogels without and with antibiotics are reversible or not. For this purpose, all disks were discarded, and the culture medium was replaced with a new one without antibiotic after 48 h of L929 cells incubation with hydrogel samples and free antibiotic. Cell survival was measured again with Alamar Blue after another 48 h. The fluorescence intensity of resorufin is directly proportional to the number of viable cells. Thus, the change in fluorescence intensity measured after 48 h and 96 h of incubation is able to assess the trend of increasing or decreasing the number of living cells in the sample after the removal of the hydrogel disk samples and free antibiotic (see Supplementary Materials, Figure S1). Thus, the number of cells in the wells increases significantly, where L929 was incubated with hydrogels without and with antibiotics at concentrations of 0.5 mg/mL and 5 mg/mL. This trend indicates the resumption of normal proliferation. According to our hypothesis, in the case of using pure hydrogels and hydrogels with a low concentration of cefazolin, the main effect is the acidification of the culture medium due to the partial release of WPI molecules. pH 6.5 is the limiting factor for cell multiplication [76]. A decrease in extracellular pH leads to a decrease in intracellular pH [77]. However, protein synthesis inside the cell requires a normal or slightly alkaline pH [78]. Therefore, the cell cycle progression was inhibited during the incubation with empty hydrogels or hydrogels containing low cefazolin concentration. However, when the source of acidification of the culture medium was removed, the cells resumed normal division.

The data for the case of incubation with hydrogels containing cefazolin at a concentration of 5 mg/mL are interesting (see Supplementary Materials, Figure S1). According to the increase in the fluorescence intensity at 96 h, it can be concluded that a part of the cell population was preserved as viable, which returned to normal proliferation after the removal of the disk. This cannot be said for cases of incubation with a hydrogel containing a high concentration of cefazolin (10 mg/mL) or free antibiotic at concentrations of 5 mg/mL and 10 mg/mL. The fluorescence intensity at 96 h for these samples drops significantly, indicating even more cell deaths compared to 48 h. Thus, the effect of the hydrogel with cefazolin 10 mg/mL and free antibiotic 5 mg/mL and 10 mg/mL is irreversible. In summary, despite the slight cytotoxicity of the hydrogels themselves, the use of hydrogels with encapsulated cefazolin at a concentration of 5 mg/mL can ensure the preservation of viable eukaryotic cells, in contrast to the use of free cefazolin at this concentration.

## 4. Conclusions

Hydrogels based on whey protein isolate with antibacterial properties have been obtained. The technique for drug loading into this type of hydrogel is simple without any loss of the drug. WPI-based hydrogels have a prolonged release profile of the loaded substance; a significant amount of the released drug was detected up to 48 h after the beginning of the experiment. Cefazolin retains its antimicrobial activity during the hydrogel preparation procedure. The amount of released antibiotic is high enough to suppress bacterial growth for 48 h for the hydrogel samples containing 5 mg/mL and 10 mg/mL of cefazolin. The antibacterial effect is manifested both in the liquid medium and on the surface of the nutrient agar. The use of WPI-based hydrogels as carriers of antibiotics makes it possible to reduce their overall cytotoxicity against normal healthy cells. In this regard, it seems promising to use WPI hydrogels with a prolonged release of the drug as a material for coating implants and manufacturing other medical devices with antibacterial properties.

## Data Availability

The data underlying the results presented in this paper are not publicly available at this time but may be obtained from the authors upon reasonable request.

## Supplementary Materials

The following are available online at https://www.mdpi.com/article/10.3390/pharmaceutics14061199/s1, Figure S1: Viability of L929 cell line incubated with hydrogel disks empty and containing cefazolin at concentration 0.5 mg/mL, 5 mg/mL, 10 mg/mL, and free antibiotic at the same concentrations for 48 h and 96 h.
