# Supplementary material for: WPI Hydrogels with a Prolonged Drug-Release Profile for Antimicrobial Therapy
Source: Pharmaceutics. 2022 Jun 4;14(6):1199. doi: 10.3390/pharmaceutics14061199 (PMC9231275; doi:10.3390/pharmaceutics14061199)
Supplement: Supplementary file 1 [file pharmaceutics-14-01199-s001.zip › pharmaceutics-1725911-supplementary.pdf]

Supplementary Materials: WPI Hydrogels with a Prolonged Drug-Release Profile for Antimicrobial Therapy

Valentina O. Plastun [1,*], Ekaterina S. Prikhozhdenko [1], Olga I. Gusliakova [1], Svetlana V. Raikova [2,3], Timothy E. L. Douglas [4,5], Olga A. Sindeeva [6] and Oksana A. Mayorova [1,7,*]

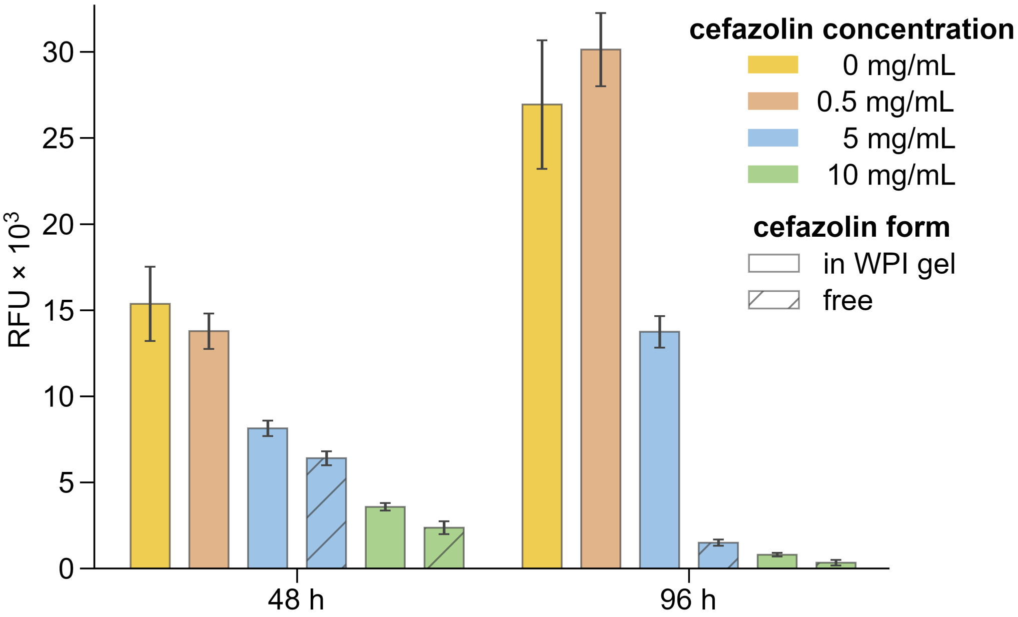

Figure S1. Viability of L929 cell line incubated with hydrogel disks empty and containing cefazolin at concentration 0.5 mg/mL, 5 mg/mL, 10 mg/mL, and free antibiotic at the same concentrations for 48 h and 96 h.
